# The Long and Winding Road: My Journey Designing Technology to Support Successful Aging

**DOI:** 10.1093/geroni/igaf122.334

**Published:** 2025-12-31

**Authors:** Wendy Rogers

**Affiliations:** University of Illinois Urbana-Champaign, Champaign, Illinois, United States

## Abstract

In the Human Factors and Aging Laboratory, which I direct, we take a broad view of successful aging. We think about it is being able to do what you want, when you want, where you want, how you want, and with whom you want. In short, the goal is autonomy, and the quest is to determine how technology can be used as an augmentative tool. We explore technology in many forms from apps on a smart phone, websites, videochat platforms, digital home assistants, to assistive robots – social, mobile, and interactive. Our approach is to think about the context in which the technology will be used and the range of users (e.g., older adults, family members, professional caregivers). We follow a user-centered design process that engages subject matter experts and end-users throughout, designing with them not for them. My degrees are all in psychology, thus my focus is always on the human side of the human-technology interaction. I think about what the person wants to do; what their limitations and preferences are; and especially what kind of support they will need to be able to take advantage of what the technology has to offer. I combine my training in cognitive psychology, human factors, experimental research methods, and qualitative assessments to develop mixed methods approaches to the research questions. I will provide examples of our research in the McKechnie Family LIFE Home that illustrate the process of fulfilling the potential for technology to support the autonomy of older adults.

